# Editorial: Addiction and social behaviors in the post-pandemic world

**DOI:** 10.3389/fpsyg.2024.1414233

**Published:** 2024-05-02

**Authors:** Iina Savolainen, Nicholas Kerry, Anu Sirola

**Affiliations:** ^1^Faculty of Social Sciences, Tampere University, Tampere, Finland; ^2^Positive Psychology Center, University of Pennsylvania, Philadelphia, PA, United States; ^3^Department of Social Sciences and Philosophy, University of Jyväskylä, Jyväskylä, Finland

**Keywords:** addiction, alcohol use and abuse, social behavior, behavioral change, COVID-19, post-pandemic

Since its onset in early 2020, the COVID-19 pandemic profoundly challenged the fabric of our daily lives, our wellbeing, and the nature of our social interactions (Vindegaard and Benros, 2020; Alizadeh et al., 2023). Initially, many responded to the pandemic by retreating into quarantine, an experience marked by social isolation and withdrawal from conventional daily interactions. As we moved into the post-pandemic phase, our society had undergone significant changes, with altered social conventions now rooted in our everyday reality.

The pandemic notably impacted health-related behaviors and those with addictive potential (Avena et al., 2021). Changes in our social interactions and entertainment during the pandemic manifested in activities such as online gaming, social media use, and broader internet consumption. While these behaviors served to maintain connections during times of isolation, there existed a risk that they would become permanent patterns, persisting even after returning to regular activities and social engagements (Zvolensky et al., 2020). This context underscored the need for research addressed in our Research Topic “*Addiction and social behaviors in the post-pandemic world*.” This collection of research on diverse populations offers insights into the nuanced ways in which the pandemic has influenced, and continues to influence, addictive and social behaviors, providing a crucial understanding of the challenges and opportunities that lie ahead.

Reilly et al.'s investigation into the pandemic experiences of US veterans established that a significant portion of veterans experienced anxiety about COVID-19, leading to an increase in the consumption of alcohol, sedatives, inhalants, tobacco, and cannabis. Even after adjusting for pre-existing problematic substance use, the negative consequences of the pandemic and self-reported feelings of loneliness due to COVID-19 were associated with worsened physical and mental health during the pandemic. Similarly, Akeman et al. found a deterioration in mental health among students during the pandemic, with symptoms worsening as the pandemic persisted. Those employing active coping strategies were less prone to symptom exacerbation, while increased alcohol consumption prior to the pandemic correlated with heightened symptoms. Additionally, Chen et al. investigated college students in the post-pandemic period, examining psychological distress, metacognitions regarding smartphone usage, and problematic smartphone usage. Their work highlighted psychological distress as a significant predictor of problematic smartphone usage and identified variations in the underlying mechanisms linking diverse forms of psychological distress to problematic smartphone usage, providing insights into prevention and intervention strategies targeting problematic smartphone usage among this population.

Online use patterns, such as social media use, changed during the pandemic (Marciano et al., 2022). Social media thrives on content creation and sharing, and Da-yong and Zhan established that individuals' personality traits play a significant role in shaping their motivations to share content, particularly in the realm of short video sharing. Highly conscientious users are inclined to share short videos for altruistic reasons rather than for image management or emotional expression, and they actively manage their online time. Conversely, extraversion and neuroticism have a robust positive influence on motivations related to image management, altruism, emotional expression, leisure, and conformity. These results provide important insight into future investigations on excessive time spent on social media and the spread of content and (mis)information. Breckwoldt et al. assessed the frequency and duration of video gaming amidst the COVID-19 pandemic among athletes, noting a significant surge in video gaming activity during the initial lockdown period, which they suggest was beneficial in helping athletes adapt to the prevailing circumstances. Kim et al. utilized a 3-year longitudinal dataset involving adolescents and examined the impact of gaming duration on psychological variables such as loneliness, depression, self-esteem, and life satisfaction. Their findings also underscored the role of social capital as a moderator, with higher levels of social capital in gaming cohorts enhancing self-esteem and life satisfaction. These results emphasize the importance of gaming in promoting coping mechanisms and social capital, particularly in situations where social interaction relies on virtual platforms. Despite the benefits of internet gaming, further research is needed to understand whether its risk of becoming an addictive behavior could outweigh these benefits (Von der Heiden et al., 2019).

The study by Bottel et al. contributed to existing knowledge of how to effectively measure problematic online activities by differentiating internet use disorder (IUD) from internet gaming disorder (IGD). By adapting the DSM-5 criteria for IGD as questions for IUD, the authors were able to detect which criteria were most significant in predicting IUD. “Loss of control”, “continued overuse”, and “mood regulation” were frequently endorsed criteria by participants who were categorized as heavy internet users, but “jeopardizing” emerged as the strongest predictor of IUD, followed by “loss of interest” and “continued overuse.” The findings provide support for potential adjustments to the DSM-5 criteria for IUD to enhance the accuracy of diagnosis of internet use disorders.

Taking a practical approach to addressing wellbeing during the post-pandemic era, Dash et al. assessed the impact of adolescent-provider connectedness on STI risk reduction 3 months after intervention across two therapeutic approaches: Motivational Interviewing and Brief Adolescent Mindfulness. Their study suggests that psychotherapeutic common factors, such as adolescent-provider connectedness, may play a crucial role in mitigating adolescent health risks in behavioral interventions targeting hazardous drinking or sexual activity. Romero Reyes et al. created a psychometric scale based on the Theory of Planned Behavior, designed to gauge attitudes, subjective norms, self-efficacy, past help-seeking behaviors, and intentions to seek help among young adults exhibiting hazardous and harmful alcohol consumption. The findings indicate that the designed instrument can contribute to developing and/or evaluating interventions that promote students' help-seeking behavior.

Overall, these nine studies underscore the complex interplay between the COVID-19 pandemic and shifts in social and addictive behaviors among different demographics. Going forward, it is critical to develop new measures and implement enhanced strategies to address the negative impact of the pandemic on mental health and addictive behaviors.

## Author contributions

IS: Writing – original draft, Writing – review & editing. NK: Writing – review & editing. AS: Writing – review & editing.
